# PacBio HiFi reads data sets for the ATCC-authenticated isolate of Candida albicans SC5314 (ATCC MYA-2876)

**DOI:** 10.1099/acmi.0.001142.v3

**Published:** 2026-08-13

**Authors:** Lois L. Hoyer, Elizabeth K. Hogan, Alvaro G. Hernandez

**Affiliations:** 1 Department of Pathobiology, College of Veterinary Medicine, University of Illinois Urbana-Champaign, Urbana, IL, USA; 2 Roy J. Carver Biotechnology Center, University of Illinois Urbana-Champaign, Urbana, IL, USA

**Keywords:** authenticated isolate, *Candida albicans*, genome sequence, PacBio HiFi reads, SC5314

## Abstract

*Candida albicans* is a yeast of major medical importance. The genome sequence for the reference isolate SC5314 has been available for many years and is displayed as a phased diploid assembly through a public community resource. As DNA sequencing technologies continue to advance, new high-quality data sets can be generated to augment existing genome resources. Here, we present Pacific Biosciences (PacBio) HiFi data sets with exceptional read length and sequencing depth. The data sets were generated from *C. albicans* SC5314 newly obtained from the American Type Culture Collection, thereby authenticating the isolate to a community-accessible source. The PacBio HiFi data set with a mean read length greater than 14 kb was assessed for its potential to support construction of a phased diploid genome assembly. Most chromosomes were effortlessly resolved as gapless, phased haplotypes with telomeres at both ends; however, challenging regions including the rDNA locus on one copy of chromosome R remained longer than could be fully resolved using these high-quality, state-of-the-art data. The PacBio HiFi reads data sets are available through GenBank and provide a valuable resource for exploring diploid loci in this important reference strain.

## Data Summary

The PacBio HiFi DNA sequence reads were deposited into the GenBank Sequence Read Archive under accession numbers SRR32357978 (9 kb mean read length) and SRR32357977 (14 kb mean read length) as part of BioProject PRJNA1224063. Deposit of phased diploid genome assemblies required separate BioProject numbers. Haplotype 1 received BioProject number PRJNA1307021 and haplotype 2 received BioProject number PRJNA1307020.

## Introduction

*Candida albicans* is a yeast of high medical relevance and the focus of considerable research effort over the past several decades [1, 2]. A key resource for investigations into *C. albicans* biology and pathogenesis is its genome sequence that is curated and displayed for the research community on the *Candida* Genome Database [3, 4]; the genome is presented as a phased diploid assembly. This long-standing resource was developed and progressively refined over many years using DNA sequencing approaches that were state of the art at the time [5–7]. Recently, we demonstrated how the current state-of-the-art DNA sequencing technology [Pacific Biosciences (PacBio) highly accurate (HiFi) long DNA sequence reads] could be used to close gaps in the reference assembly and extend the chromosome sequences to the telomeres on its eight chromosome pairs [8]. We presented this work as a collapsed haploid genome assembly (ASM3268872v1) and provided detailed methods for using it as a template to explore the PacBio HiFi reads file (SRR23724250) for any genomic region of interest.

While considering use of SRR23724250 as the foundation for development of an improved *C. albicans* SC5314 phased diploid genome assembly, we reflected on the importance of rigorous authentication of the SC5314 isolate. Concerns about the reproducibility of research findings have been prominent for at least the past decade [9]. In response to this ‘Replication Crisis’, authentication of research resources has become a common requirement in grant applications [10], reflecting broader efforts to improve reproducibility across laboratories. The American Type Culture Collection (ATCC; Manassas, VA) provides *C. albicans* strain SC5314, enabling researchers to obtain an authenticated isolate for experimental use. Information available on the ATCC website, together with correspondence from an ATCC technical support representative, documents that *C. albicans* strain SC5314 was deposited into the ATCC collection on 20 December 2002 [11, *2025 private communication (e-mail) from American Type Culture Collection technical support to the author*]. The available chain-of-custody information suggests strong links between this deposited strain, the *C. albicans* genome project and the sequence data represented on the *Candida* Genome Database. An improved genome assembly and annotation for SC5314 should be rigorously authenticated against an isolate that is traceable, stable and accessible to other researchers.

This line of thinking follows the trajectory for development of the *Saccharomyces cerevisiae* reference genome (strain S288C) [12, 13]. *S. cerevisiae* was the first eukaryote to have a completely sequenced genome [12]. Producing this genome assembly required effort from many research groups internationally, and although focused on strain S288C, included data from various *S. cerevisiae* isolates [13]. The resulting genome assembly was groundbreaking, yet a chimaera that did not completely correspond to any one of the isolates used to derive it. Engel *et al*. [13] described this fascinating history and the decision in 2010 to revise the S288C reference assembly using data collected from a single yeast colony.

It is common knowledge in the *Candida* research community that there can be considerable variation between isolates called ‘SC5314’ in different research laboratories yet this widely accepted idea does not seem to be documented in the scientific literature. The first author of this paper recalls a presentation by Prof. Frank Odds at a *Candida* and Candidiasis conference that demonstrated variation among isolates called SC5314. Prof. Odds contacted *Candida* researchers worldwide and requested they send him their SC5314 isolate. He applied a molecular fingerprinting technique to the isolates and coded the results to avoid embarrassment for individual researchers when he revealed obvious – even shocking – variation among the ‘SC5314’ isolates he collected. There are many potential causes for variation among the isolates including the remarkable plasticity of the *C. albicans* genome [14], differences in preservation and handling of *C. albicans* isolates between research laboratories and the likelihood that some of the isolates were not SC5314 in the first place. All these reasons provide a compelling argument for establishing the *C. albicans* SC5314 genome sequence from a single colony from an authenticated source.

Although our laboratory’s isolate of SC5314 was obtained from the investigators who later deposited the strain into the ATCC collection, it was acquired before the 20 December 2002 deposit date. This laboratory isolate was used to generate SRR23724250 and ASM3268872v1. Although our SC5314 has been handled with considerable care, it cannot be rigorously authenticated against a community-accessible reference source such as ATCC. We therefore purchased SC5314 from ATCC, immediately plated the culture, selected a single representative colony and used that colony to generate the PacBio HiFi reads data sets that are the focus of this Technical Resource. Publishing the data sets as a Technical Resource enhances the likelihood that they will be discovered and used by the *Candida* research community to provide authenticated sequence information from any genomic region of interest.

## Methods

*C. albicans* strain SC5314 (ATCC MYA-2876) was newly purchased from the ATCC (Manassas, VA) for the focused purpose of deriving genome sequence data. Upon arrival, the isolate was streaked to a YPD agar plate (per litre: 10 g yeast extract, 20 g Bacto peptone, 20 g dextrose, 20 g Bacto agar) and incubated at 30 °C in an air incubator. A representative isolated colony was inoculated into 50 ml YPD medium in a 250 ml Erlenmeyer flask. The flask was incubated at 30 °C with 200 r.p.m. shaking for 16 h. Aliquots of the culture were placed into sterile cryovials, and sterile glycerol was added to a final concentration of 38%. The cryovials were stored at −80 °C. The remainder of the culture was used for extraction of high-molecular-weight genomic DNA for long-read DNA sequencing [15].

Two independent data sets are reported here. For one, the genomic DNA was sheared to an average length of 13 kb using a Megaruptor 3 system (Diagenode) then converted to a library using the SMRTbell Prep Kit 3.0 (Pacific Biosciences). For the second, the genomic DNA was sheared to an average length of 6 kb with PowerSoil beads and converted into a library using the HiFi Plex Prep Kit 96. Libraries were size-selected for fragments of 3–50 kb on a BluePippin system with a 0.75% gel cassette and DNA marker S1 (Sage Science). Libraries were sequenced on a Revio single-molecule real-time (SMRT) cell on a PacBio Revio system using a Revio Polymerase Kit with standard primer, the circular consensus sequencing (CCS) mode, and a 30 h movie time. CCS and demultiplexing analysis used SMRT Link v13.1 (ccs --min-passes 3 --min-rq 0.99).

Because longer sequence reads provide greater benefit for assembling the *C. albicans* genome, the data set derived from 13 kb average length genomic DNA was assessed for its ability to readily support assembly of a gapless, telomere-bounded, phased diploid genome sequence. Filtlong v0.2.1 [16] was used to select reads of at least 14 kb (–min_length 14,000). This process reduced the sequence coverage from ~630× to 367×. The length-filtered reads were assembled into haplotypes using hifiasm v0.19.6 with default parameters [17]. SnapGene v8.1.1 (https://www.snapgene.com/) was used for general manipulation of sequence files.

## Technical resource descriptions

PacBio Revio HiFi reads created from SC5314 genomic DNA sheared to an average length of 13 kb were deposited into GenBank under Sequence Read Archive accession number SRR32357977. The data set had 638,971 reads with a mean length of 14,413 bp. The data set included over 9.2 billion bp of sequence information (~630× genome coverage). The longest read was 47,903 bp with N_50_=15,124 bp.

PacBio Revio HiFi reads created from SC5314 genomic DNA sheared to an average length of 6 kb were deposited into GenBank under Sequence Read Archive accession number SRR32357978. The data set had 724,783 reads with a mean length of 9,392 bp. The data set included over 6.8 billion bp of sequence information (~460× genome coverage). The longest read was 32,346 bp with N_50_=10,358 bp.

Although the PacBio HiFi reads data sets are the focus of this Technical Resource, we explored their capacity to readily construct a gapless, telomere-bounded, phased diploid genome assembly. The hifiasm programme was selected because it was originally designed for PacBio HiFi reads and focuses on preserving contiguity of haplotypes [17]. The resulting haplotype 1 included 121 contigs. Of these, 9 contigs had telomeric repeat sequences at one or both ends, consistent with chromosome-length assembly (Table 1). Other contigs only included rDNA sequences (71 total) or mitochondrial genome sequences (40 total). One haplotype 1 contig (40,693 bp) was found entirely on chromosome 4 in haplotype 2. Haplotype 2 included 13 contigs, 9 of which had telomeric repeats at one or both ends. The other four contigs had only rDNA repeats. Contigs that were only rDNA repeats, mitochondrial genome sequences, or contained entirely on an assembled chromosome were not examined further.

**Table 1.** Details for the draft phased diploid *C. albicans* SC5314 genome assembly that can be produced readily from the SRR32357977 data set

Chr*	Haplotype 1^†^	Haplotype 2^†^
1	**T** 3,234,266 T	**T** 3,210,278 **T**
R (rDNA)	**T** 1,980,827 (NNN) 443,003 T	**T** 1,953,264 (NNN)
2	**T** 2,265,996 **T**	**T** 2,253,858 **T**
3	**T** 1,796,691 (NNN)	**T** 1,796,855 (NNN)
4	**T** 1,638,378 **T**	**T** 1,641,412 **T**
5	**T** 1,259,422 **T**	**T** 1,238,716 **T**
6	**T** 1,072,355 **T**	**T** 1,035,907 **T**
7	**T** 969,790 **T**	**T** 607,586 (NNN) 396,260 **T**
Total	14,660,728	14,134,136
N_50_	1,980,827	1,953,264
L_50_	3	3

*The standard nomenclature for *C. albicans* chromosomes numbers them from 1 to 7, largest to smallest [19]. Since the size of the rDNA-containing chromosome can change markedly due to expansion/contraction of the rDNA repeat copy number, it was named R to recognize its unpredictable size [19].

†Chromosome sizes are shown in bp. **T** indicates the presence of telomeric repeats on that end of a chromosome. (NNN) indicates where 100 N were added to join two large contigs or indicate an incomplete chromosome. NNN were not included in the calculated genome size for each haplotype. Individual contigs were gapless and did not contain any N except those indicated by (NNN).

Compared to the intensive process needed to generate the initial phased diploid genome assembly available on the *Candida* Genome Database [3–7], state-of-the-art, long-read data can readily produce a credible draft genome assembly. Reads could be extracted from the data set to join the two chromosome 7 fragments in haplotype 2 and to complete the < 2,000 bp missing from the right ends of both chromosome 3 haplotypes. In contrast, breakage of the chromosome R homologs likely reflects the extensive rDNA repeat array present in SC5314. The true repeat copy number may exceed the length of the longest reads in the HiFi data sets, suggesting that even-longer-read DNA sequencing technologies will be needed to resolve this genomic region completely.

The HiFi reads data sets can be converted into blast databases using blast+ [18] and queried. The draft phased diploid assembly is included among the Technical Resources to serve as a roadmap for exploring the HiFi reads data sets and facilitating extraction of authenticated sequence information for genome regions of interest.

## Abbreviations

ATCC: American Type Culture Collection
CCS: circular consensus sequencing
PacBio: Pacific Biosciences
SMRT: single-molecule real-time
YPD: yeast extract–peptone–dextrose

## References

[1] Clancy CJ, Calderone RA. (2014). Candida and Candidiasis.

[2] Odds FC (1988). Candida and Candidosis: a Review and Bibliography.

[3] Skrzypek MS, Binkley J, Binkley G, Miyasato SR, Simison M (2017). The *Candida* Genome Database (CGD): incorporation of Assembly 22, systematic identifiers and visualization of high throughput sequencing data. Nucleic Acids Res.

[4] CGD *Candida* Genome Database. https://www.candidagenome.org.

[5] van het Hoog M, Rast TJ, Martchenko M, Grindle S, Dignard D (2007). Assembly of the *Candida albicans* genome into sixteen supercontigs aligned on the eight chromosomes. Genome Biol.

[6] Muzzey D, Schwartz K, Weissman JS, Sherlock G (2013). Assembly of a phased diploid *Candida albicans* genome facilitates allele-specific measurements and provides a simple model for repeat and indel structure. Genome Biol.

[7] Butler G, Rasmussen MD, Lin MF, Santos MAS, Sakthikumar S (2009). Evolution of pathogenicity and sexual reproduction in eight *Candida* genomes. Nature.

[8] Hoyer LL, Freeman BA, Hogan EK, Hernandez AG (2024). Use of a *Candida albicans* SC5314 PacBio HiFi reads dataset to close gaps in the reference genome assembly, reveal a subtelomeric gene family, and produce accurate phased allelic sequences. Front Cell Infect Microbiol.

[9] Collins FS, Tabak LA (2014). Policy: NIH plans to enhance reproducibility. Nature.

[10] National Institutes of Health (NIH) (2017). Reminder: Authentication of Key Biological and/or Chemical Resources. https://grants.nih.gov/grants/guide/notice-files/NOT-OD-17-068.html.

[11] American Type Culture Collection (ATCC) *Candida albicans* (Robin) Berkhout, ATCC MYA-2876 (Strain SC5314). https://www.atcc.org/products/mya-2876.

[12] Fisk DG, Ball CA, Dolinski K, Engel SR, Hong EL (2006). *Saccharomyces cerevisiae* S288C genome annotation: a working hypothesis. Yeast.

[13] Engel SR, Dietrich FS, Fisk DG, Binkley G, Balakrishnan R (2014). The reference genome sequence of *Saccharomyces cerevisiae*: then and now. G3.

[14] Vande Zande P, Zhou X, Selmecki A (2023). The dynamic fungal genome: polyploidy, aneuploidy and copy number variation in response to stress. Annu Rev Microbiol.

[15] Hoyer LL (2023). Extraction of yeast high-molecular-weight genomic DNA v1. Protocols.io.

[16] Wick RR, Menzel P (2019). Filtlong. https://github.com/rrwick/Filtlong.

[17] Cheng H, Concepcion GT, Feng X, Zhang H, Li H (2021). Haplotype-resolved de novo assembly using phased assembly graphs with hifiasm. Nat Methods.

[18] National Center for Biotechnology Information (NCBI) Blast+. https://blast.ncbi.nlm.nih.gov/doc/blast-help/downloadblastdata.html.

[19] Wickes B, Staudinger J, Magee BB, Kwon-Chung KJ, Magee PT (1991). Physical and genetic mapping of *Candida albicans*: several genes previously assigned to chromosome 1 map to chromosome R, the rDNA-containing linkage group. Infect Immun.

